# Distribution of proteinase K‐resistant anti‐α‐synuclein immunoreactive axons in the cardiac plexus is unbiased to the left ventricular anterior wall

**DOI:** 10.1111/pin.13389

**Published:** 2023-12-01

**Authors:** Nei Fukasawa, Miku Maeda, Yoshifumi Sugiyama, Takahiro Fukuda, Masayuki Shimoda

**Affiliations:** ^1^ Department of Pathology The Jikei University School of Medicine Tokyo Japan; ^2^ Division of Clinical Epidemiology, Research Center for Medical Sciences The Jikei University School of Medicine Tokyo Japan; ^3^ Division of Community Health and Primary Care, Center for Medical Education The Jikei University School of Medicine Tokyo Japan; ^4^ Medical Center for Memory & Cognitive Disorders Sasebo Chuo Hospital Nagasaki Japan

**Keywords:** α‐synuclein, cardiac plexus, Lewy body disease

## Abstract

Lewy body disease (LBD) is characterized by the appearance of Lewy neurites and Lewy bodies, which are predominantly composed of α‐synuclein. Notably, the cardiac plexus (CP) is one of the main targets of LBD research. Although previous studies have reported obvious differences in the frequency of Lewy body pathology (LBP) in the CP, none of them have confirmed whether LBP preferably appears in any part of the CP. Thus, we aimed to clarify the emergence and/or propagation of LBP in the CP. In this study, 263 consecutive autopsy cases of patients aged ≥50 years were included, with one region per case selected from three myocardial perfusion areas (MPAs) and subjected to proteinase K and then immunohistochemically stained with anti‐α‐synuclein antibodies to assess LBP. We stained all three MPAs in 17 cases with low‐density LBP and observed the actual distribution of LBP. LBP were identified in the CP in 20.2% (53/263) of patients. Moreover, we found that LBP may appear in only one region of MPAs, mainly in the young‐old group (35.3% (6/17) of patients). These findings suggest that it is possible to underestimate LBP in the CP, especially in the young‐old group, by restricting the search to only one of the three MPAs.

**Abbreviations**
AαS: anti‐aggregated‐α‐synuclein
CNS: central nervous system
CP: cardiac plexus
DLB: dementia with Lewy body
ILBD: incidental Lewy body disease
IR: immunoreactive
LAD: left anterior descending artery region
LBD: Lewy body disease
LBP: Lewy body pathology
LBs: Lewy bodies
LCX: left circumflex artery region
LVAW: left ventricular anterior wall
MPA: myocardial perfusion area
NIA‐AA: National Institute on Aging–Alzheimer's Association
PAF: pure autonomic failure
PD: Parkinson's disease
PK: proteinase K
PKRαS: proteinase K‐resistant anti‐α‐synuclein
PαS: anti‐phosphorylated‐α‐synuclein
RCA: right coronary artery region
*SD*
: standard deviation
αS: α‐synuclein

## INTRODUCTION

Lewy body disease (LBD) refers to a group of neurodegenerative diseases characterized by Lewy body pathology (LBP), Lewy neurites, and Lewy bodies (LBs). To date, LBD includes Parkinson's disease (PD), dementia with Lewy bodies (DLB), PD with dementia, pure autonomic failure, and incidental LBD (ILBD).^1^, ^2^, ^3^, ^4^, ^5^, ^6^, ^7^, ^8^, ^9^, ^10^, ^11^, ^12^, ^13^

The major component of LBP is α‐synuclein (αS), a 140‐amino acid protein located in the nucleus and presynaptic terminal.^14^, ^15^, ^16^, ^17^ αS, which exists either as a soluble monomer that lacks a specific secondary structure or as a cell membrane with an α‐helical structure,^18^, ^19^ functions as a molecular chaperone that is involved in neurotransmitter release at neural synapses and vesicle recycling.^20^ Misfolding of αS has been linked to degenerative processes, which include conversion from monomers to oligomers, the formation of oligomers with proteinase K (PK) resistance, and the self‐assembly of amyloid fibrils.^21^, ^22^, ^23^, ^24^ The dual‐hit hypothesis^25^, ^26^, ^27^, ^28^ suggests that the abnormal self‐organization of αS initially occurs in the peripheral nervous system, through environment exposure, and then ascends, prion‐like, to the central nervous system (CNS). However, reports have shown that LBD localizes to the internal organs and structures, such as the cardiac plexus (CP) and sympathetic ganglia, which implies that the lesions are multi‐centric in origin.^29^, ^30^, ^31^, ^32^, ^33^, ^34^, ^35^ Given its frequent involvement in LBD, the cardiovascular system has been a common diagnostic target using methods such as (123)I‐meta‐iodobenzylguanidine cardiac scintigraphy and histopathological studies (see Supporting Information: Table S1).

However, only a few studies including a substantial number of asymptomatic patients have reported on the incidence of LBP in the CP.^35^, ^36^ Moreover, various studies have reported bias in terms of the site of cardiac tissue sampling, particularly the anterior left ventricular wall.^35^, ^37^, ^38^, ^39^, ^40^, ^41^, ^42^, ^43^, ^44^, ^45^ Therefore, the current study aimed to calculate more reliable frequencies of LBP in CP from consecutive autopsy cases and to provide recommendations for more appropriate sampling sites based on the assessment of its distribution.

## MATERIALS AND METHODS

### Patients

Tissue samples were obtained from consecutive autopsies performed in patients aged ≥50 years at the Department of Pathology, Jikei University Hospital between January 2011 and December 2020. From the 272 initial autopsies performed during this period, nine were excluded due to Creutzfeldt–Jakob disease in two patients, constrictive pericarditis with severe calcification in one patient, absence of heart dissection in two autopsies, loaning of paraffin block specimens to other institutions in another two autopsies, severe drying and degeneration of the tissues before paraffin embedding in one patient, and lack of clinical information in one patient. The exclusion of these cases was not dependent on the presence of clinicopathological LBD. Thus, tissues for analysis were obtained from the remaining 263 autopsies, with CNS samples (i.e., samples from brain and spinal cord) available from 93 autopsies. Information from the medical records and autopsy reports was collected without knowledge of LBP status in the CP.

### Ethics approval and consent to participate and for publication

In this study, all procedures involving human participants were conducted in accordance with the ethics standards of the institutional and/or national research committee and with the 1964 Helsinki Declaration and its later amendments or comparable ethics standards. This study was approved by the Ethics Committee of Jikei University Hospital (approval number: 33–035 (10645)). Given the retrospective observational nature of this study with an opt‐out format, patient consent was not required.

### Heart specimens

The hearts were cut in short‐axis at the time of autopsy and fixed in 10% buffered neutral formalin solution (Muto Pure Chemicals, Tokyo, Japan) or 10% formalin (Mitsubishi Gas Chemical, Tokyo, Japan). Notably, the heart specimens were collected from the cut surface near the base of the heart. The size of the specimen was approximately 40 × 25 mm. The median postmortem time was 9.7 h (range: 1.0–143.0 h, standard deviation (SD) 14.7 h), and the median formalin fixation time was 32 days (range: 1–1747 days, SD: 282 days). Among the formalin‐fixed paraffin‐embedded blocks of the heart tissue, one specimen was selected per case from any three myocardial perfusion areas (MPAs), as mentioned below, of the left ventricular wall containing a large number of nerve fascicles. After selection, a pathologist not involved in the histological examination determined the MPAs to which the heart tissue belonged. Based on the gross images and gross descriptions of the heart, blocks including the posterior wall and septum of the left ventricle were considered blocks from the right coronary artery region (RCA), those including the anterior wall and septum of the left ventricle were considered blocks from the left anterior descending artery region (LAD), and those belonging to the lateral wall distant from the septum were considered blocks from the left circumflex artery region (LCX).

### Immunohistochemistry and evaluation

After deparaffinization, 4‐µm‐thick sections cut from each block were treated with PK (160 µg/mL in 10 mmol/L Tris‐HCl, pH 8.0) for 30 min and then immunostained using a cocktail of anti‐αS antibodies (PK‐resistant anti‐αS (PKRαS): anti‐αS 1–10, rabbit, polyclonal, COSMO BIO, Tokyo, Japan, Catalog No. TIP‐SN‐P01, dilution ratio 1:5000; anti‐αS 131–140, rabbit, polyclonal, COSMO BIO, Tokyo, Japan, Catalog No. TIP‐SN‐P09, dilution ratio 1:5000). Additionally, 4‐µm‐thick sections were immunostained using anti‐aggregated‐αS antibody (AαS) (mouse, monoclonal, EMD Millipore, Temecula, CA, USA, Catalog No. MABN389, Clone No. 5G4, dilution ratio 1:5000) and anti‐phosphorylated‐αS antibody (PαS) (mouse, monoclonal, Wako, Tokyo, Japan, Catalog No. 014‐20281, Clone No. pSyn#64, dilution ratio 1:10 000), without PK treatment.

Histological and immunohistochemical evaluations were performed by two pathologists based on light microscopy observations (Eclipse Ni‐U, Nikon, Tokyo, Japan; BX53, Olympus, Tokyo, Japan). Further, PKRαS‐, AαS‐, and PαS‐immunoreactive (IR) axons were considered as LBP.

Finally, we mapped PKRαS‐IR axons on slides to semi‐quantitatively assess their frequency of appearance and categorize them into four groups (Groups A–D). Notably, in the semi‐quantitative analysis, we classified all cases in comparison to the representative cases of each group (as presented in the Comparison of CP LBP among four groups subsection of the Results). For Group C, all three MPAs were stained, which allowed us to assess the actual distribution of PKRαS‐IR axons. Additional analyses for Group C were performed to identify cases in which PKRαS‐IR axons were not found in one or two of the three regions of MPAs (i.e., in cases that could be false‐negatives when only one MPA was sampled). For this purpose, we searched all three regions in Group C because we expected to observe a clear difference in the density of distribution between the three regions in Group C, which had a lower distribution density.

### CNS specimens

The tissues of the CNS (including the brain and spinal cord and, in addition, the olfactory bulb and stellate ganglion) were fixed in 10% buffered neutral formalin solution (Muto Pure Chemicals, Tokyo, Japan) or 10% formalin (Mitsubishi Gas Chemical, Tokyo, Japan) for about 2 weeks and representative sites were embedded in paraffin. Then, 6‐µm‐thick sections were cut and stained with hematoxylin and eosin, Klüver‐Barrera, Gallyas‐Braak, and Bodian methods. Immunohistochemistry was also performed for PαS, cocktail of polyclonal anti‐amyloid β antibodies (rabbit, polyclonal, Immuno‐Biological Laboratories, Gumma, Japan, Catalog No. 18580, dilution ratio 1:500; rabbit, polyclonal, Immuno‐Biological Laboratories, Gumma, Japan, Catalog No. 18582, dilution ratio 1:500), anti‐phosphorylated paired helical filament tau (mouse, monoclonal, Thermo Fisher Scientific, Rockford, IL, USA, Catalog No. MN1020, Clone No. AT8, dilution ratio 1:10 000) and anti‐phosphorylated Tar deoxyribonucleic acid binding protein of 43 kDa (rabbit, polyclonal, COSMO BIO, Tokyo, Japan, Catalog No. TIP‐PTD‐P02, dilution ratio 1:10 000).

### Statistical analyses

Statistical analyses were performed using JMP version 16 (SAS Institute Inc., Cary, NC, USA). Comparisons between the PKRαS‐IR positive and ‐negative groups were made using Welch's *t*‐test and χ^2^ test. A *P*‐value of <0.05 indicated statistical significance.

## RESULTS

### Patient information

The mean age of the patients at death was 72.8 years (range: 50–117 years, SD: 11.3 years). Overall, 193 men (73.4%) and 70 women (26.6%) were included in the study. Of them, 59 (22.4%) had severe inflammation, which was histopathologically defined as follows: infection or abscess in multiple organs, infection or abscess in soft tissues disseminated via the bloodstream, splenitis, hemophagocytosis, and diffuse alveolar damage in the exudative phase. Notably, advanced solid tumors or hematopoietic/lymphoid tissue tumors were found in 115 patients (43.7%). These conditions (severe inflammation and/or tumors) were the cause of death in most patients. Myocardial infarction was the most common cardiovascular disease (71 cases (27.0%)), followed by valvular disease (22 cases (8.4%)). Of the 31 cases (11.8%) in which surgery or intravascular intervention was performed, the coronary artery was targeted in 22 cases (71.0%). CNS samples were obtained from 93 of 263 patients (35.4%). Meanwhile, ischemic lesions, intracranial hemorrhage, and moderate‐to‐severe arteriosclerosis were detected in 52 cases (55.9%), 21 cases (22.6%), and 69 cases (74.2%), respectively. According to the National Institute on Aging–Alzheimer's Association (NIA–AA)'s ABC scoring criteria, the degree of neuropathologic change indicative of Alzheimer's disease was judged to be ‘Not’ or ‘Low’ in 83 cases (89.2%).^46^, ^47^ The following conformational diseases were also observed: amyloid angiopathy, 17/93 (18.3%); argyrophilic grain disease, 13/93 (14.0%); amyotrophic lateral sclerosis/frontotemporal lobar degeneration, 3/93 (3.2%); and progressive supranuclear palsy, 1/93 (1.1%).

One patient was diagnosed with Parkinson's syndrome before death and was suspected to have DLB. And in this case, Lewy‐related disease subtype^9^ was ‘Amygdala‐predominant’ and the degree of neuropathologic change indicative of AD was judged to be ‘Intermediate’. No other patients were suspected as having LBD.

### Morphological alterations in LBP found in the CP

**Figure 1:**
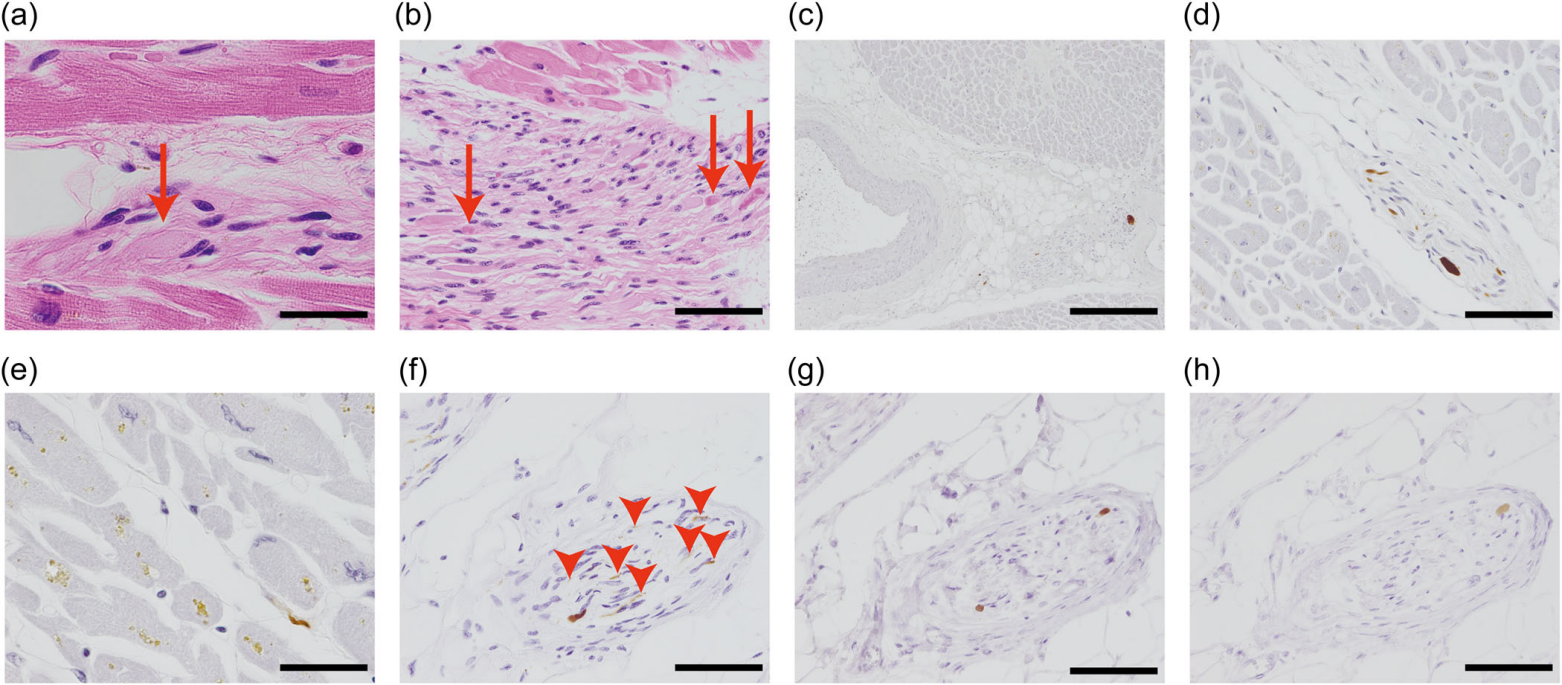
Representative images of Lewy body pathology in the cardiac plexus. (a, b) Nerve fascicles distributed from the epicardium to the outer myocardium in hematoxylin and eosin‐stained sections. Swollen axons (a: arrow) containing pale acidophilic amorphous material were observed (pale bodies), with some of them having a strongly acidophilic core (b: arrows) (LBs). (c–e) Most PKRαS‐IR axons were observed as enlarged nerve axons in the nerve fascicles around coronary arterial branches and were distributed in the epicardium or outer myocardium (c, d). Perimysial positive axons (dots or short threads) were rarely observed (e). (f–h) PKRαS‐IR (f), AαS‐IR (g), and PαS‐IR images (h) of the same nerve fascicle in the serial sections. PKRαS‐IR images showed more axons with less conspicuous swollen axons than the those shown by AαS‐IR and PαS‐IR images (arrow heads). Scale bar = 20 μm (a, e), 50 μm (b, d, f–h) and 200 μm (c). AαS‐IR = anti‐aggregated‐α‐synuclein immunoreactive; LBs, Lewy bodies; PKRαS‐IR = proteinase K‐resistant anti‐α‐synuclein immunoreactive; PαS‐IR = anti‐phosphorylated‐α‐synuclein immunoreactive. [Color figure can be viewed at wileyonlinelibrary.com]

Hematoxylin and eosin staining revealed swollen axons containing pale acidophilic amorphous material (pale bodies) among the nerve fascicles distributed from the epicardium to the outer myocardium (Figure 1a), with some having a strongly acidophilic core (LBs) (Figure 1b).

PKRαS‐IR axons were observed as clusters of enlarged nerve axons in the nerve fascicles around the coronary artery branches and were mostly distributed in the epicardium or outer myocardium (Figure 1c, d). In 10 cases, PKRαS‐IR perimysial nerve axons (dots or short threads) (Figure 1e) were observed. In two of the 10 cases, only a few PKRαS‐IR perimysial nerve axons were identified. Based on the evaluation of the same nerve fascicle in serial sections, PKRαS‐IR axons were found to be more frequent and detailed than AαS‐IR and PαS‐IR axons (Figure 1f–h).

### Comparison of CP LBP among four groups

**Figure 2:**
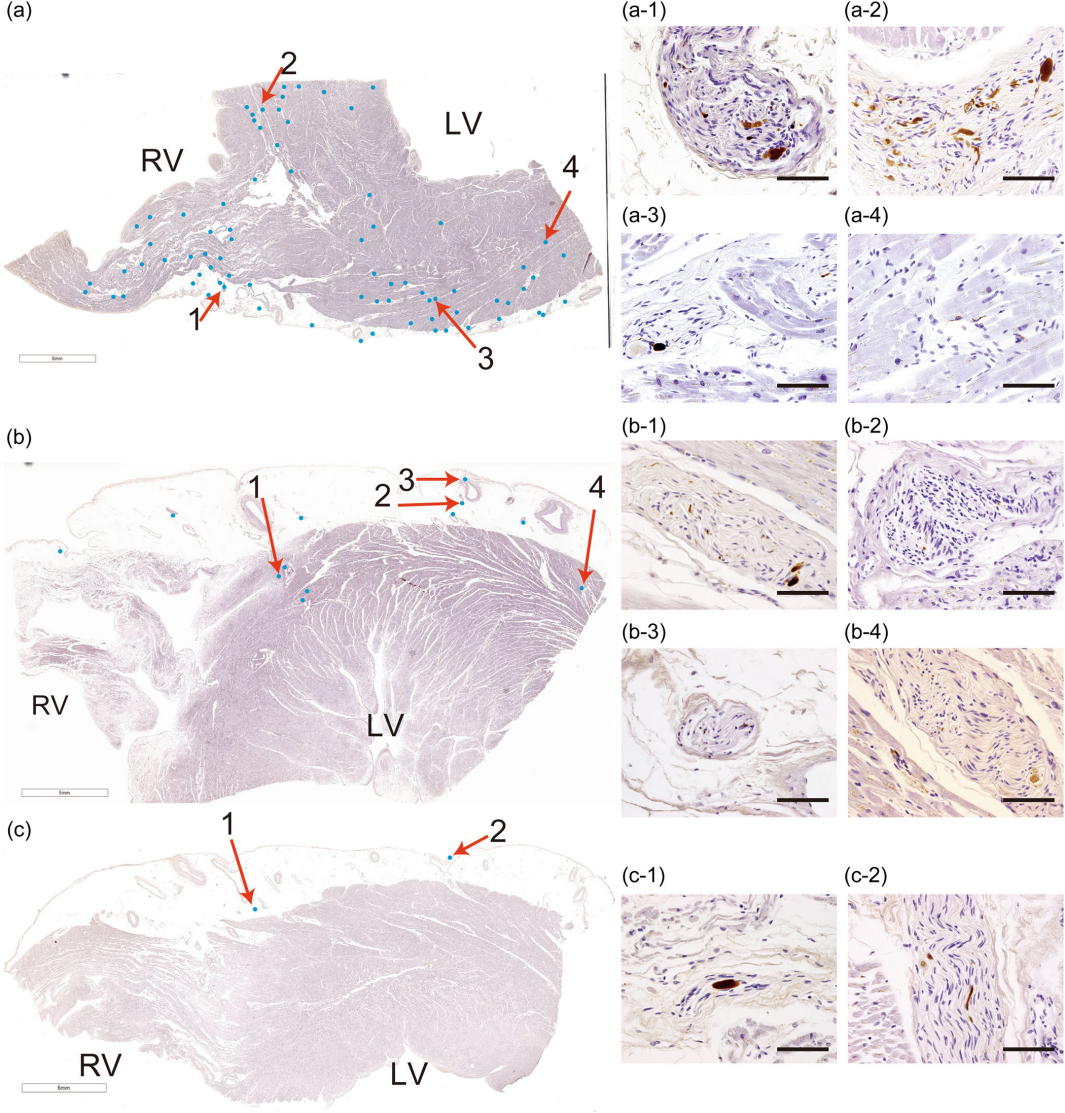
Representative cases in the semi‐quantitative assessment of Lewy body pathology distribution density. (a–c) Representative cases classified as Group A (a), Group B (b), and Group C (c) in the semi‐quantitative evaluation are presented. Cases exhibiting similar distributions and densities on these images were categorized into three groups. Group D comprised cases in which no PKRαS‐IR axons were identified in the selected slides. Images of axons belonging (a) to the right coronary artery region (b) and (c) to the left anterior descending artery region. The blue dots in each of the three loupe images indicate the position of the observed PKRαS‐IR axon. High magnification images of the numbered blue dots are shown in the respective numbered images (a‐1–4, b‐1–4, and c‐1–2). In panel (a), multiple PKRαS‐IR axons formed clusters within perivascular nerve fascicles in the epicardium (a‐1). Multiple PKRαS‐IR axons also formed clusters within nerve fascicles in the deep septum (a‐2) and myocardium (a‐3). In addition, perimysial PKRαS‐IR axons were identified (a‐4). In panel (b), the nerve fascicles containing PKRαS‐IR axons were found near the boundary between the myocardium and epicardium (b‐1 and 4) and in the epicardium (b‐2 and 3), showing a clear distribution bias toward the epicardium compared with panel (a). In panel (c), the distribution trend of PKRαS‐IR axons was similar to that shown in panel (b), but the number of PKRαS‐IR axons was <5 in the entire slide. Scale bar = 50 μm (a‐1–4, b‐1–4, and c‐1–2). LV = left ventricle; PKRαS‐IR = proteinase K‐resistant anti‐α‐synuclein immunoreactive; RV = right ventricle. [Color figure can be viewed at wileyonlinelibrary.com]

PKRαS‐IR axons were mapped for the semi‐quantitative assessment of LBP distribution density. The representative images of Groups A–C are presented in Figure 2. We categorized the cases exhibiting similar distributions and densities on these images as follows. Group D comprised cases in which no PKRαS‐IR axons were identified in the selected slides. Group A comprised cases in which multiple PKRαS‐IR axons formed clusters within perivascular nerve fascicles in the epicardium (Figures 2a‐1). Notably, multiple PKRαS‐IR axons also formed clusters within nerve fascicles in the deep septum (Figures 2a‐2) and myocardium (Figure 2a‐3). In addition, perimysial PKRαS‐IR axons were identified (Figure 2a‐4). Group B comprised cases in which the nerve fascicles containing PKRαS‐IR axons were found near the boundary between the myocardium and epicardium (Figures 2b‐1 and 4) and in the epicardium (Figures 2b‐2, 3), showing a clear distribution bias toward the epicardium compared with that in Group A. Group C comprised cases in which the distribution trend of PKRαS‐IR axons was similar to that in Group B, but the number of PKRαS‐IR axons was <5 on the entire slide.

### Demographic distribution of PKRαS‐positive patients in all subjects and in the three MPAs

The PKRαS‐positive (53 cases; 20.2%) and ‐negative (210 cases; 79.8%) groups had a mean age of 77.4 years (range: 50–97 years, SD: 11.5 years) and 71.6 years (range: 50–117 years, SD: 10.4 years), respectively (Table 1). The mean age of the PKRαS‐positive group was significantly higher than that of the negative group (*P* = 0.0014). Among the 193 men and 70 women, 37 and 16 (37/193: 19.2% and 16/70: 22.9%) were PKRαS‐positive, respectively, with no significant difference between the two groups (*P* = 0.5101). Among the 53 PKRαS‐positive patients, 38 (38/263: 14.4%) and 35 (35/263: 13.3%) were positive for AαS and PαS, respectively. AαS‐ and/or PαS‐IR axons were not observed in PKRαS‐negative patients.

**Table 1:** Proteinase K‐resistant anti‐α‐synuclein immunoreactive axons in the cardiac plexus of 263 patients did not preferably appear in a specific myocardial perfusion area.

	Overall *N* = 263	PKRαS‐IR axons in the cardiac plexus					
		Positive (Groups A–C) *n* = 53 (20.2)				Negative (Group D) *n* = 210 (79.8)	*P*‐value
			Group A *n* = 16 (30.2)	Group B *n* = 19 (35.8)	Group C *n* = 18 (34.0)		
Mean age (SD)	72.8 (11.3)	**77.4 (11.5)**	77.0 (8.2)	77.3 (11.9)	77.9 (13.2)	**71.6 (10.4)**	0.0014*
Sex, male/female	193 (73.4)/70 (26.6)	**37 (69.8)/16 (30.2)**	9 (56.3)/7 (43.8)	11 (57.9)/8 (42.1)	17 (94.4)/1 (5.6)	**156 (74.3)/54 (25.7)**	0.5101†
RCA	43 (16.4)	**10**	5	1	4	**33**	0.3467†
LAD	54 (20.5)	**14**	5	6	3	**40**	
LCX	166 (63.1)	**29**	6	12	11	**137**	

LAD = left anterior descending artery region; LCX = left circumflex artery region; PKRαS‐IR = proteinase K‐resistant anti‐α‐synuclein immunoreactive; RCA = right coronary artery region; SD = standard deviation.

*P*‐values were calculated using the values for positive and negative cases (given in bold). Under line: *P* < 0.05.

Data are presented as *n* (%) unless otherwise indicated.

* Welch's *t*‐test.

^†^ χ^2^ test.

The MPAs to which all 263 heart tissues belonged were as follows: RCA region, 43 patients (16.4%), mean age = 72.2 years (range: 51–97 years, SD: 11.5 years), male to female ratio = 31:12, and PKRαS‐IR‐positive to PKRαS‐IR‐negative ratio = 10:33; LAD region, 54 patients (20.5%), mean age = 73.3 years (range: 50–117 years, SD: 13.0 years), male to female ratio = 40:14, and PKRαS‐IR‐positive to PKRαS‐IR‐negative ratio = 14:40; and LCX region, 166 patients (63.1%), mean age = 72.7 years (range: 50–98 years, SD: 10.7 years), male to female ratio = 122:44, and PKRαS‐IR‐positive to PKRαS‐IR‐negative ratio = 29:137. There was no bias in the frequency of PKRαS among the three regions of MPA (*P* = 0.3467).

### Young‐old group cases without PKRαS‐IR axons distributed in the LAD region

Semi‐quantitative assessment of PKRαS‐IR axon density showed that among the 53 positive cases, 16 (30.2%), 19 (35.8%), and 18 (34.0%) were categorized into Groups A, B, and C, respectively. The mean age of Groups A–C was around 77 years, which was almost the same as that for all positive cases.

In Group C, all three regions of MPAs were stained. Notably, Group C included 18 cases, but excluded one case in which only one region was originally sampled. Age and sex by the number of MPAs in which PKRαS‐IR axons could be identified were as follows (Table 2): three positive areas = eight cases (8/17: 47.1%, mean 82.5 years, range: 67–89 years, SD: 6.6, male to female ratio 7:1); two positive areas = three cases (3/17: 17.6%, mean: 84.0 years, range: 69–97 years, SD: 11.5, male to female ratio = 3:0); one positive area = six cases (two RCA and four LCX; 6/17: 35.3%; mean: 67.2 years, range: 50–94 years, SD: 14.9, male to female ratio = 6:0). Cases with PKRαS‐IR axons identified in only one of the three MPAs had a lower mean age and belonged to the young‐old group.

**Table 2:** Patient information of 17 cases in Group C searched for proteinase K‐resistant anti‐α‐synuclein immunoreactive axons in the all three MPAs.

	Overall	The number of MPAs that were positive for PKRαS‐IR axons		
		3 areas	2 areas	1 area
*n* (%)	17 (100.0)	8 (47.1)	3 (17.6)	6 (35.3)
Mean age (range, SD)	77.4 (50–97, 13.4)	82.5 (67–89, 6.6)	84.0 (69–97, 11.5)	67.2 (50–94, 14.9)
Sex, male/female	16/1	7/1	3/0	6/0
MPAs with identified PKRαS‐IR axons			RCA + LAD: 1 case	RCA: 2 cases
			RCA + LCX: 1 case	
			LAD + LCX: 1 case	LCX: 4 cases

LAD = left anterior descending artery region; LCX = left circumflex artery region; MPA = myocardial perfusion area; PKRαS‐IR = proteinase K‐resistant anti‐α‐synuclein immunoreactive; RCA = right coronary artery region; SD = standard deviation.

Observation of PαS‐IR structures in 93 CNS sampled cases revealed abnormal αS in the CNS or CP in 21 cases (Table 3). Of these, 11 cases (Cases 1–11: Group A, three cases; Group B, six cases; Group C, two cases) exhibited abnormal αS in the CNS and CP. In the two cases in Group C (Cases 10 and 11), PKRαS‐IR axons were identified in all three MPA regions. Six cases (Cases 12–17: Group A, one case; Group B, three cases; Group C, two cases) exhibited abnormal αS mainly in the CP. Of the two Group C cases (Cases 16 and 17), one exhibited PKRαS‐IR axons confined to the RCA and one exhibited PKRαS‐IR axons confined to the LCX. In four cases (Cases 18–21), abnormal αS was found in the CNS and no PKRαS‐IR axons were observed in the CP.

**Table 3:** Distribution of Lewy body pathology in 21 patients with abnormal α‐synuclein in the CP and/or CNS.

Case	Age/sex Lewy‐related disease subtypes	CP	SG	SC	Brainstem regions			Basal forebrain/limbic regions					Neocortical regions			OB
		Groups A‐D (PαS‐IR axons positive/negative)			dmV/irx	LC/R	SN	nbM	Amyg	CA2	TEC	GC	T cx	F cx	P cx	
1^a^	Late 60s/M	A (positive)	3	Rare LNs	Rare LNs	0	Rare LNs	0	0	0	0	0	0	0	0	0
2	Early 70s/M Brainstem‐predominant	A (positive)	0	1	2	3	2	2	Rare LNs	Rare LNs	Rare LNs	Rare LNs	0	0	0	ND
3^b^	Mid 80s/F	A (positive)	ND	1	ND	ND	ND	1	ND	0	0	0	0	0	0	ND
4	Mid 70s/M Brainstem‐predominant	B (negative)	3	1	3	3	1	1	1	0	0	0	0	0	0	ND
5	Late 70s/M Brainstem‐predominant	B (positive)	ND	1	3	2	1	2	2	Rare LNs	0	0	0	0	0	2
6	Early 70s/F Brainstem‐predominant	B (negative)	3	2	3	3	2	2	1	Rare LNs	Rare LNs	1	0	0	0	0
7	Late 70s/F Limbic (transitional)	B (positive)	ND	2	2	2	1	2	2	0	1	1	1	1	0	Rare LNs
8	Early 80s/F Brainstem‐predominant	B (positive)	ND	Rare LNs	1	1	2	2	ND	1	1	1	0	0	0	0
9^c^	Late 80s/F Amygdala‐predominant	B (positive)	2	Rare LNs	1	2	1	1	3	Rare LNs	1	1	1	Rare LNs	Rare LNs	1
10	Mid 80s/M Diffuse neocortical	C: All three MPAs (negative)	3	2	3	3	2	3	2	Rare LNs	Rare LNs	2	Rare LNs	1	1	2
11	Mid 80s/M Brainstem‐predominant	C: All three MPAs (positive)	0	0	2	1	1	2	ND	Rare LNs	Rare LNs	1	Rare LNs	0	0	0
12	Mid 70s/F	A (positive)	ND	ND	0	0	0	0	0	0	0	0	0	0	0	ND
13	Mid 50s/M	B (positive)	2	0	0	0	0	0	0	0	0	0	0	0	0	0
14	Late 90s/M	B (positive)	ND	0	0	0	0	0	0	0	0	0	0	0	0	0
15	Mid 50s/F	B (negative)	1	ND	0	0	0	0	0	0	0	0	0	0	0	ND
16	Early 60s/M	C: RCA only (negative)	ND	ND	0	0	0	0	0	0	0	0	0	0	0	Rare LNs
17	Mid 70s/M	C: LCX only (negative)	0	0	0	0	0	0	0	0	0	0	0	0	0	0
18	Mid 60s/M Brainstem‐predominant	D (negative)	2	1	2	2	1	1	Rare LNs	Rare LNs	0	0	0	0	0	1
19	Mid 70s/M Brainstem‐predominant	D (negative)	0	0	Rare LNs	1	1	0	1	0	1	0	0	0	0	Rare LNs
20	Mid 80s/M Brainstem‐predominant	D (negative)	3	1	2	2	1	1	1	Rare LNs	Rare LNs	0	0	0	0	1
21	Mid 50s/F Brainstem‐predominant	D (negative)	ND	Rare LNs	Rare LNs	Rare LNs	Rare LNs	0	0	0	0	0	0	0	0	ND

Amyg = amygdala; CA2 = cornu Ammonis of hippocampus, region 2; CP = cardiac plexus; dmV/irx = dorsal motor nucleus of the vagus/intermediate reticular zone; F = female; F cx = frontal cortex; GC = gyrus cinguli; LC/R = locus coeruleus/raphe; LCX = left circumflex artery region; LNs = Lewy neurites; M = male; nbM = nucleus basalis of Meynert; ND = no data; OB = olfactory bulb; P cx = parietal cortex; PαS‐IR = anti‐phosphorylated‐α‐synuclein antibody immunoreactive; RCA = right coronary artery region; SC = spinal cord; SG = stellate ganglion; SN = substantia nigra; T cx = temporal cortex; TEC = transentorhinal cortex.

The results evaluated by proteinase K‐resistant anti‐α‐synuclein antibody and anti‐phosphorylated‐α‐synuclein antibody for cardiac plexus and anti‐phosphorylated‐α‐synuclein antibody for central nervous system are presented.

Lewy‐related disease subtypes are presented in McKeith et al.^9^

Semi‐quantitative analysis of alpha‐synuclein deposition was performed according to that presented in McKeith et al.^10^

^a^ This case was reported in Fukasawa et al.^29^

^b^ In this case, the brainstem was inadequately sampled due to the collapse of CNS tissue caused by total cerebral ischemia.

^c^ This case was diagnosed with Parkinson's syndrome before death (mentioned in ‘Patient information’).

## DISCUSSION

The current study primarily showed that the frequency of LBP in the CP could be limited to approximately 20% and that MPAs might have caused a bias in the appearance of LBP in the young‐old group.

Among the included cases, 20.2% (53/263) showed PKRαS positivity in CP, whereas 14.4% (38/263) exhibited AαS and/or PαS positivity in CP. AαS‐ and/or PαS‐positive cases were frequently found only in PKRαS‐positive cases. Previous studies have suggested that the conformational transition to the stable PKRαS oligomer is an early rate‐limiting step leading to fibril formation in the evolution of abnormal αS.^22^ Moreover, research has shown that phosphorylation of αS is characteristic to LBP and has aggregation‐inhibitory and neuroprotective properties.^48^, ^49^ Our findings showed that although PKRα‐IR axons were abnormally swollen, they were still illustrated better than AαS‐ and/or PαS‐IR axons. Hence, we believe that PKRαS‐IR axons correspond to PKRαS oligomers and beyond and that the emergence of AαS‐ and/or PαS‐IR axons likely indicates a mature abnormal αS amyloid structure. Several previous studies on LBP in CP have been conducted in symptomatic patients with PD, DLB, or pure autonomic failure^29^, ^30^, ^31^, ^32^, ^33^, ^34^, ^35^, ^36^, ^37^, ^38^, ^39^, ^40^, ^41^, ^42^, ^43^, ^44^, ^45^, ^50^, ^51^, ^52^, ^53^, ^54^, ^55^, ^56^ (see Supporting Information: Table S1). In two of such studies, Navarro‐Otano et al.^36^ and Tanei et al.^35^ examined LBP in the CP of cohorts that included many non‐symptomatic patients, with large discrepancies in their results (7.7%, 7/91^36^ and 18.9%, 98/518^35^) according to our calculations of their data. Our results were similar to those reported by Tanei et al. Although differences in staining methods may be responsible, AαS‐ and/or PαS‐axons were identified in 14.4% of the overall population in this study, which was also double that reported by Navarro‐Otano et al. The upper limit for the frequency of LBP in CP of autopsied cases is likely to be approximately 20%. Navarro‐Otano et al. mainly examined tissues obtained during cardiovascular surgery. In contrast, our study and that of Tanei et al. examined autopsy cases. Therefore, we considered that the patient background in these studies was more diverse than that in Navarro‐Otano et al.'s study. Thus, patient background—especially the presence of severe inflammation—may explain the significant difference in the frequency of abnormal αS positivity in our study and Tanei et al.'s study compared with Navarro‐Otano et al.'s study, although further investigation is required to confirm this.

The current study selected heart blocks regardless of MPAs of the three coronary arterial branches and did not limit our sampling site to the left anterior descending artery region/left ventricular anterior wall (LAD/LVAW). There was no bias in the frequency of PKRαS among the three regions of MPA (*P* = 0.3467). Sympathetic nerves in the heart are widely distributed throughout the nerve fascicles of the epicardium and between myocardial cells. In fact, previous studies have revealed that LBP is distributed from the epicardial adipose tissue to the muscular layer,^29^, ^30^, ^31^, ^43^, ^44^, ^45^, ^50^, ^51^, ^54^ and this distribution was also observed in the current study. In many previous studies, sampling sites were limited to specific regions of the heart. In particular, several studies have used the LAD/LVAW as the sampling sites.^35^, ^37^, ^38^, ^39^, ^40^, ^41^, ^42^, ^43^, ^44^, ^45^ This phenomenon has been attributed to the greater distribution of sympathetic nerves in the LAD/LVAW than in the posterior wall, as well as the high uptake of (123)I‐meta‐iodobenzylguanidine cardiac scintigraphy in the region.^38^, ^57^ However, no studies have shown that LBP in the heart always involves the LAD/LVAW or is restricted to a specific region. In Group C, which had a lower distribution density, nine of the 17 cases (52.9%) had no PKRα‐IR axons in at least one MPA, whereas six cases (35.3%: two RCA and four LCX) had PKRαS‐IR‐positive axons in only one region. Therefore, when the distribution density was low, a variation existed in the occurrence of LBP among MPAs, which could lead to false negative results when the sampling site is limited. In our study, the mean age of the positive group was 77.6 years and was significantly higher than that of the negative group. Tanei et al. reported that the frequency of LBP increased with age, whereas Navarro‐Otano et al. reported that those ≥70 years had a higher frequency of LBP in the CP. In contrast, the mean age of the aforementioned six cases is clearly lower (67.2 years) than that reported by Tanei et al. and Navarro‐Otano et al. Although the participants of our study were suggested to have incidentally confirmed LBD, most of them died due to other illnesses, making it difficult to obtain data on the presence of nonmotor symptoms. Therefore, it is unclear whether these six cases reflect the early stages of LBD. However, for the younger‐old group, we believe that sampling focused on only one region of the three MPAs would cause underestimation for assessing LBP in CP. Among the cases in which PKRαS‐IR axons were mainly in CP, both cases from Group C exhibited PKRαS‐IR axons in only one region of MPAs. When searching for LBPs localized to the heart, we recommend to search three MPAs.

In conclusion, our results suggest that all three regions of MPAs should be evaluated when discussing the initial emergence and frequency of LBP. Furthermore, sampling should not be limited to the short‐axis of the heart; instead, efforts should be made to obtain information regarding the long‐axis.

## LIMITATIONS OF THIS STUDY

Given that this was a cross‐sectional study of autopsy cadavers, clinical information other than that available could not be obtained and causal relationships could not be explored. Specifically, data on many nonmotor symptoms thought to precede LBD, such as constipation and rapid eye movement sleep behavior disorder, or exogenous factors, such as previous infections, drugs, and occupational exposure, were often lacking. A larger longitudinal study including histopathological examination is needed to resolve these issues. Finally, the formalin fixation time was not uniform, which may have affected detection of abnormal αS.

## AUTHOR CONTRIBUTIONS

Nei Fukasawa analyzed and interpreted histological examinations and patient data and was a major contributor to the manuscript. Miku Maeda collected clinical information and determined the MPA to which the heart blocks belonged. Yoshifumi Sugiyama contributed to the statistical analysis. Takahiro Fukuda contributed to the histological examination and editing of the manuscript. Masayuki Shimoda contributed to the editing of the manuscript. All authors read and approved the final manuscript.

## CONFLICT OF INTEREST STATEMENT

Masayuki Shimoda is an Editorial Board member of *Pathology International* and a co‐author of this article. To minimize bias, they were excluded from all editorial decision‐making related to the acceptance of this article for publication.

## Supporting information

Supporting information.

## Data Availability

The data that support the findings of this study are available from the corresponding author upon reasonable request.
