# Supplementary material for: Distribution of proteinase K‐resistant anti‐α‐synuclein immunoreactive axons in the cardiac plexus is unbiased to the left ventricular anterior wall
Source: Pathol Int. 2023 Dec 1;74(1):1–12. doi: 10.1111/pin.13389 (PMC11551827; doi:10.1111/pin.13389)
Supplement: Supplementary file 1 — Supporting information. [file PIN-74-1-s001.docx]

# **Supplementary Material**

## **Table S1:** Studies on the cardiac plexus, sympathetic ganglia, and Lewy body disease in organs localized in a closed environment

|  | **Author (published year)** | **Antibodies, dilution ratio** | **Sampling sites within the heart** | **Symptomatic LBD/Overall (n)** | **Age (range, mean)** | **Sex (male/female)** | **LBP n = αS-positive/sample size (%)** | | |
| --- | --- | --- | --- | --- | --- | --- | --- | --- | --- |
|  |  |  |  |  |  |  | **PNS** | | **CNS** |
|  |  |  |  |  |  |  | **CP** | **SG** |  |
| Studies that observed the distribution of LBs and/or LNs | | | | | | | | | |
|  | Wakabayashi et al. (1993)^56^ | not used | Unclear | 12/195 (ILBD: 7) | ND | ND | ND | ND | 19/195 (9.7) |
|  | Wakabayashi et al. (1997)^55^ | not used | Unclear | PD: 30 | ND | ND | 12/30 (40.0) |  |  |
|  |  |  |  | PD: 30 | ND | ND |  | 28/30 (93.3) |  |
|  |  |  |  | non-PD: 60 | ND | ND |  | 5/60 (8.3) |  |
|  | | | | | | | | | |
| Studies that showed denervation/dysfunction of immunohistochemically tyrosine hydroxylase-positive nerve fibers in the cardiac plexus | | | | | | | | | |
|  | Orimo et al. (2002)^37^ | not use | LVAW | 5/13 | 55−91, 70.5 | 9/4 | ND | ND | 5/13 (38.5) |
|  | Orimo et al. (2005)^38^ | PαS, 1:5000 | LVAW | 22/50 | 55−92, (76.0) | 23/27 | ND | ND | 23/50 (46.0) |
|  | Amino et al. (2005)^39^ | not use | LVAW | 4/9 | 70−93, 85.1 | 3/6 | ND | ND | 4/9 (44.4) |
|  | Orimo et al. (2007)^40^ | PαS (psyn#64), 1:5000 | LVAW | 0/30 (ILBD: 20) | 55−87, 70.7 | 19/11 | ND | 14/30 (46.7) | 20/30 (66.7) |
|  | Dickson et al. (2008)^41^ | αS (NACP98), 1:5000 | LVAW | 16/45 (ILBD: 12) | 62−97, 77.4 | 32/13 | ND | ND | 28/45 (55.6) |
|  | | | | | | | | | |
| Studies with fewer than 10 subjects involving the heart or SG | | | | | | | | | |
|  | Polvikoski et al. (2006)^31^ | αS, ND | Unclear | 0/1 (ILBD: 1) | 89 | F | + | + (main) | + |
|  | Miki et al. (2009)^30^ | αS, ND | Unclear | 0/1 (ILBD: 1) | 35 | M | + | + (main) | - |
|  | Mori et al. (2012)^32^ | αS, ND | Unclear | 0/1 (ILBD: 1) | 86 | M | - | + (main) | + |
|  | Fukasawa et al. (2018)^29^ | PKRαS, AαS, PαS | Both atria and ventricles | 0/1 (ILBD: 1) | 67 | 1/0 | + | + (main) | + |
|  | Orimo et al. (2001)^42^ | αS (NACP-5), 1:1000 | LVAW | 1/3 | 57−84, 67.0 | ND | 0/3 (0) | 1/3 (33.3) | 1/3 (33.3) |
|  | Mitsui et al. (2006)^43^ | PαS (psyn#64), ND PαS (PSer129), ND | LVAW | 1/6 | ND | ND | ND | 1/6 (16.7) | 1/6 (16.7) |
|  | Nishida et al. (2017)^54^ | PαS (LB508), 1:500 | Both atria and ventricles | 0/2 (ILBD: 2) | 68, 68.0 | 2/0 | 2/2 (100.0) | ND | 2/2 (100.0) |
|  | | | | | | | | | |
| Studies with more than 10 subjects involving the heart or SG | | | | | | | | | |
|  | Iwanaga et al. (1999)^53^ | αS, 1:500 | LV RA | 11/57 (ILBD: 7) | 46−87, 67.1 | ND | 16/57 (28.1) | 14/57 | 18/57 (31.6) |
|  | Orimo et al. (2008)^44^ | PαS (pSyn#64), 1:5000 | LVAW | 10/60 (ILBD: 20) | 55−92, 70.7 | 36/24 | 24/60 (40.0) | 23/50 (46.0) | 31/60 (51.7) |
|  | Ghebremedhin et al. (2009)^52^ | αS (syn-1), 1:2000 | LVAW RVAW Sinus node Posterior intercaval Atrial area | 5/14 (ILBD: 2) | 62−86, 74.9 | 8/6 | 5/14 (35.7) | ND | 7/14 (50.0) |
|  | Fujishiro et al. (2008)^45^ | αS (NACP98), 1:3000 | LVAW | 14/29 (ILBD: 11) | 62−95, 77.4 | 8/21 | 21/29 (72.4) | ND | 25/29 (86.2) |
|  | Beach et al. (2010)^50^ | PαS (PSer129), 1:80 | LV RV Epicardium of heart At the apex | 45/92 (ILBD: 7) | 38−99, 82.5 | 55/37 | 1/42 (2.4)  0/44 (0) | 14/38 (36.8)  19/56 (33.9) | 50/89 (56.2) |
|  | Gelpi et al. (2014)^51^ | αS (KM51), 1:500 PαS (psyn#64), 1:1000 | Multiple areas | 15/28 (ILBD: 5) | 62−93, 81.4 | 9/19 | 16/28 (57.1) | 16/28 (57.1) | 20/28 (71.4) |
|  | | | | | | | | | |
| Studies involving a large number of non-LBD cases | | | | | | | | | |
|  | Navarro-Otano et al. (2013)^36^ | αS (KM51), 1:500 PαS (psyn#64), 1:1000 | Multiple areas | 91 (ILBD: 7) | 31−84, 67.0 | 62/29 | 7/91 (7.7) | ND | ND |
|  |  |  | LV | 14 (ILBD: 1) | 46−85, 68.0 | 6/8 | 1/14 (7.1) | ND | ND |
|  | Tanei et al. (2021)^35^ | PαS (psyn#64), 1:20000 PαS (PSer129), 1:100 PαS (MJF-R13), 1:80000 αS (LB509), 1:100 | LVAW | 46/518 (ILBD: 132) | 24-111, 81.0 | 303/215 | 98/518 (18.9) | 125/518 (24.7) | 169/518 (32.4) |
|  | | | | | | | | | |
| Other studies on LBD localized in a closed environment | | | | | | | | | |
|  | Wakabayashi et al. (1999)^33^ | αS (NACP-1), 1:500 | ND | 1 (ILBD: 1) | 68 | F | ND | ND | + (brainstem) |
|  | Fumimura et al. (2007)^34^ | PαS (psyn#64), ND PαS (PSer129), ND αS (LB509), ND | ND | 56/783 (ILBD: 150) | 48-104, 80.7 | 455/328 | ND | AG: 87/783 (11.1) | 206/783 (26.3) |
|  | | | | | | | | | |
|  | This report | PKαS | Random sites | 1/263 (ILBD: 52) | 50-117 (72.8) | 193/70 | 53/263 (20.2) | ND | ND |

Abbreviations: AG, adrenal gland; CNS, central nervous system; CP, cardiac plexus; ILBD, incidental Lewy body disease; LBP, Lewy body pathology; LV, left ventricle; LVAW, left ventricular anterior wall; ND, data not available or not calculable; PD, Parkinson’s disease; PNS, peripheral nervous system; RA, right atrium; RV, right ventricle; RVAW, right ventricular anterior wall; SG, sympathetic ganglia (cervical to thoracic)

Data on anti-αS antibodies, age (range and mean), male and female numbers, and frequency of LBD-related findings were obtained directly or calculated to the best extent possible from the respective paper. Although ILBD is commonly defined as the absence of symptoms indicating the presence of LBD before death, some papers limit the site of LBP to the central nervous system, whereas others extend it to other parts of the body. In this section, all cases who developed LBP in the CNS or PNS at autopsy were counted as ILBD cases. Wakabayashi et al. (1993)^56^: Sampling sites within the heart included the interatrial groove and myocardium, at least. Wakabayashi et al. (1997)^55^: The paper made no mention of whether or not the cases were from the same population in the search for each body site. Some data from Wakabayashi et al. (1993)^56^ were used. Beach et al. (2010)^50^ and Tanei et al. (2021)^35^ used PK treatment. Gelpi et al. (2014)^51^ selected multiple areas within the heart and mentioned that LBP appeared most frequently in the anterior and lateral wall of the LV.
